# Melatonin improves development of early mouse embryos impaired by actinomycin-D and TNF-α

**Published:** 2014-12

**Authors:** Behrooz Niknafs, Ahmad Mehdipour, Amaneh Mohammadi Roushandeh

**Affiliations:** 1 *Drug Applied Research Center, Tabriz University of Medical Sciences, Tabriz, Iran.*; 2 *Department of Anatomical Sciences, School of Medicine, Hamadan University of Medical Sciences, Hamadan, Iran.*

**Keywords:** *Melatonin*, *Embryo*, *Actinomycine*-*D*, *TNF-α*, *Development*

## Abstract

**Background:**

Melatonin, a reactive oxygen species (ROS) scavenger and an antioxidant, has been shown that can inhibit apoptosis. Administration of melatonin may improve embryo development in assisted reproductive technology (ART).

**Objective:**

The aim of this study was to evaluate the role of melatonin in inhibition of spontaneous and induced apoptosis by Tumor Necrosis Factor Alph (TNF-α) and actinomycin-D during preimplantation development of mouse embryos.

**Materials and Methods:**

Female BALB/c mice were superovulated with pregnant mare serum gonadotropin (PMSG) followed by human chorionic gonadotropin (HCG), then allowed to mate with male mice. The resultant 2-cell embryos were divided into six groups as follows: control (group I), melatonin (group II), actinomycin-D (group III), actinomycin-D + melatonin (group IV), TNF-α (group V), and TNF-α + melatonin (group VI). We recorded the numbers and developmental rates of the 4-cell, 8-cell, morula and blastocyst embryos. Blastocysts were stained with acridine orange in order to assess for the embryo quality.

**Results:**

The group IV showed a significantly higher developmental rate of blastocysts compared to group III (p<0.05). The number of dead blastomers was significantly decreased in group IV in comparison to group III (p<0.05). Both V and VI groups had a lower developmental rate and lesser quality of blastocysts compared with group I. There was no significant difference in the developmental rate of blastocysts from group II compared to group I (p<0.05).

**Conclusion:**

Supplementation of embryo culture media with melatonin can improve the quality and developmental rate of embryos. Melatonin can prevent cell death that was induced by TNF- α and actinomycine-D.

## Introduction

Melatonin is synthesized in the pineal gland. Its secretion is dependent upon the light/dark cycle, where its highest level occurs at night. Melatonin plays a key role in immunity, and also it has anti-tumor and antioxidant activity. Melatonin is a free radical scavenger and has antioxidant propertises (1, 2). Studies have shown that melatonin affects reproductive function. In particular, the effects of melatonin have been considered in the development of preimplantation embryos in the reproductive system (3-6). 

Reactive oxygen species (ROS) may cause poor embryo quality and delay in embryo development. A reduced level of ROS is important in the embryo culture system in order to obtain a high grade embryo in infertility clinics. Melatonin crosses all cellular membranes and penetrates into subcellular organelles such as the mitochondria and nuclei (6, 7). Melatonin preserves mitochondrial function and reduces mitochondrial oxidative stress, then inhibits subsequent apoptosis (7, 8). Some recent studies have shown that melatonin has anti-oxidant and antiapoptotic capacities by improving the development of sheep, pigs, bovine, and mouse embryos (3, 4, 6, 9, 10).

Melatonin also increases the development of embryos to the blastocyst stage (5, 6, 9, 10). The free radical scavenging and antioxidant capacity of melatonin depend on melatonin concentrations that are used during in vitro development of embryos (9, 11, 12). However, there are several reports on melatonin apoptosis capacity. As high concentrations of melatonin can induce apoptosis of cancer cells (13). Therefore, the effects of melatonin remain controversial

In this research, apoptosis was induced by Tumor Necrosis Factor Alpha (TNF-α) and actinomycin-D. The ability of TNF- α and actinomycin-D to induce apoptosis in preimplantation embryos has been reported (14). Higher concentrations of TNF- α in serum and follicular fluid have been found in cases of polycystic ovary syndrome and endometriosis (15, 16).

Furthermore, actinomycin-D alters DNA transcription function, resulting in apoptosis (17). The main aim of this study was to characterize the role of melatonin in inhibition of spontaneous and induced apoptosis by TNF-α and actinomycine-D during preimplantation development of mouse embryos.

## Materials and methods

This research was an experimental study. It was done in Drug Applied Research Center in Tabriz University of medical sciences. The procedures were approved by Committee of Medical Research Ethics of Tabriz University of Medical Sciences.

**Superovulation and oocyte collection**

The female BALB/c mice were used that weighed 25 g for collection of 2-cell embryos. The mice were inbred. Male and female BALB/c mice were purchased from Tabriz University of Medical Sciences’ animal laboratory and housed in 12:12 hr light/dark cycle at approximately 24^o^C. Five mice were studied in each group for superovulation; each female mouse received an intraperitoneal injection of 10 IU pregnant mare serum gonadotropin (PMSG; Sigma). 

At 47-49 hr following administration of PMSG, an intraperitoneal injection of 10 I.U. human chorionic gonadotropin (HCG; Merck-Serno Co.) was administered. Immediately after the HCG administration, each female mouse was mated with one male mouse. The morning after mating, mice were checked for the presence of a vaginal plug for confirmation of mating. At 42-48 hr after the HCG injection, female mice were sacrificed by cervical dislocation. Their fallopian tubes were extracted and transferred to Ham’s F-10 culture medium (Sigma Co.) under mineral oil (Sigma Co.). 2-cell embryos were released from the fallopian tube under stereomicroscope (Olympus, Japan) at sterile conditions. 

**Embryo development**

The 2-cell embryos were collected and transferred in a 250 µl droplet of Ham’s F-10 medium that contained 5% BSA as a base medium under mineral oil. Embryos were incubated at 37^o^C and 5% CO_2_ and they were divided into the following groups. The control group (group I) contained base medium (Ham’s F-10 plus 5% BSA) in 50 µl droplets. The melatonin group as group II consisted of base medium and melatonin (10 µMol; Sigma, Co.) in 50 µl droplets (8). The medium was freshly prepared. After 72 hr, the embryos were transferred into base medium. The actinomycin-D group as group III consisted of base medium plus actinomycin-D (10 ng/ml, Sigma Co.) in 50 µl droplets, which was freshly prepared (14). 

After 4 hr of cultivation, the embryo culture medium was washed and replaced with base medium. The melatonin+ actinomycin-D group as group IV consisted of embryos treated with actinomycin-D for 4 hr which the medium was replaced with medium that contained melatonin. Embryos were cultured in melatonin for 72 hr, and then transferred to base medium. The TNF-α group as group V consisted of base medium that contained TNF-α (500 ng/ml; Sigma Co.) in 50 µl droplets. This solution was freshly prepared (14). After a 72 hr cultivation period, the medium was replaced with base medium. The melatonin + TNF-α group as group VI, embryos were treated and cultured with a combination of melatonin and TNF-α medium for 72 hr which they were transferred into base medium droplets. The total embryo culture time for each of the groups was 96 hr. We recorded the numbers of embryos and their stages every 24 hr.

**Morphological assessment**

Blastocysts were stained with acridine orange (2.5 µg/ml) that was diluted in PBS for 3-4 min at room temperature and then washed again in PBS droplets (18, 19). Stained blastocysts were transferred on a slide and observed under fluorescent microscope (Olympus, Japan). The blastomers of each blastocyst based on pycnosis and density of cytoplasm were counted as dead cells. 

**Statistical analysis**

Statistical analysis was carried out using the statistical package for the social science (SPSS software version 16.0 for windows, Chicago, IL, USA). Results are expressed as percentage (Mean±SD). The percentage of each group was calculated as developmental rate. The significance of the difference between the treatments was assessed using Chi-Square test. P<0.05 was considered statistically significant.

## Results

Quantitatively, the results showed development of embryos from the 2-cell embryo to blastocysts in each of the assessed 24 hr time periods until 96 hr. Despite the increased number of 4-cell embryos in the group II compared to the group I, there were no significant differences between two groups (p<0.05). A significant decrease in development of embryos from the 2-cell to 4-cell was seen in the groups III and V (p<0.05). Actinomycin-D increased the developmental block at the 2-cell embryos, followed by a decline in the development of 4-cell embryos. There were significant differences between group III with groups I and II (p<0.05). 

Application of melatonin prevented the effects of actinomycin-D on embryo development, which was going to be as the group I. TNF-α decreased the development of 2-cell to 4-cell embryos. There were significant differences between group V with groups I and II (p<0.05). Additionally, melatonin increased the development of 2-cell embryos into 4-cell embryos (Table I). It was not observed any significant differences in the development of 2-, 4-, and 8-cell embryos and in the morula after 48 and 72 hr (data not shown).

**Figure 1:**
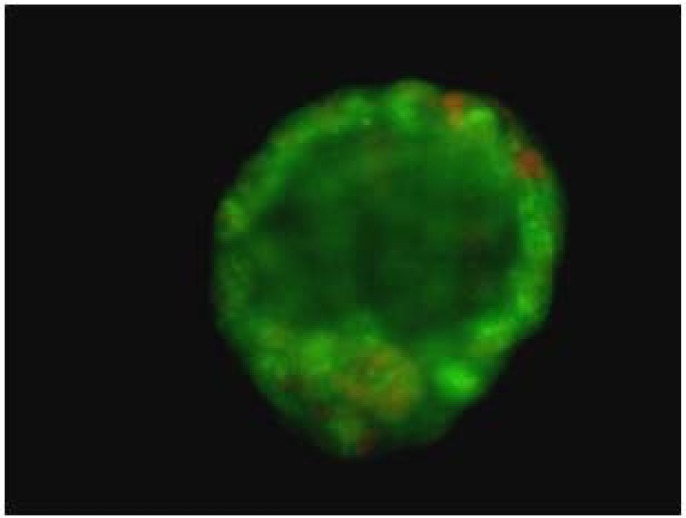
Flourecent photography of normal blastocyst with intact inner cell mass and cytotrophoblast cells

**Figure 2:**
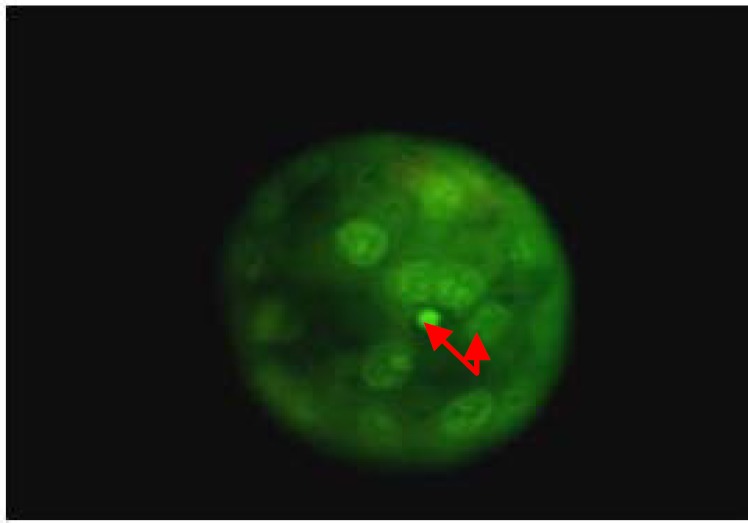
Fluorescent photography of a treated blastocyst with pycnontic (long arrow) and non-pycnotic cells (short arrow).

Blastocyst formation had decreased in groups III, V and VI after 96 hr. Among these groups, blastocyst formation was the least in group III. Although addition of melatonin improved the harmful effects of the actinomycin-D, it did not improve the development of embryos in the group V. The groups of V and VI showed significant differences in blastulation compared to group II (p<0.05) (Table II). There were significant differences between the groups I and II regards to death cells in the blastocysts (p<0.05). Actinomycin-D decreased the numbers of viable cells compared with the other groups. Melatonin increased the numbers of viable cells in blastocysts in the group II (p<0.05). There were no significant differences in the numbers of nonviable cells in groups V and VI (p<0.05) (Table II), (Figure 1, 2).

**Table I:** Comparison of embryo development between different groups after 24 hours

**Groups**	**2- cell stage (%)**	**4- cell stage (%)**	**8- cell stage (%)**
I (n=93)	37.6	54.8	7.5
II (n=98)	30.6	64.3	5.1
III (n=98)	51 b	37.8 ab	11.2
IV (n=103)	38.8	51.5	9.7
V (n=102)	46.1 b	38.2 ab	15.7
VI (n=101)	41.6	48.5 b	9.9

Data are presented as (%).

Chi-Square, p<0.05.

a : it was significant compared with group I.

b : it was significant compared with group II.

**Table II:** Comparison of embryo development and alive cells in blastocysts between different groups after 96 hours

**8**	**2- cell stage**	**4 -cell stage**	**8 -cell stage**	**Morulla**	**Blastocysts**	**Alive cells in blastocysts**
I (n=93)	35.5	22.6	11.8	9.7	20.4	64.3
II (n=98)	26.5	25.5	9.2	15.3	23.5	84.7 a
III (n=98)	39.8	30.6	10.2	10.2	9.2 ac	58.6
IV (n=103)	33	24.3	15.5	6.8	20.4 b	78.3 ab
V (n=102)	37.3	26.5	17.6	6.9	11.8 c	68.4
VI (n=101)	34.7	26.7	17.8	9.9	10.9 c	68.5

Data are presented as (%).

Chi-Square, p<0.05

a : it was significant compared with group I.

b : it was significant compared with group III.

c : it was significant compared with group II.

## Discussion

This study demonstrated that melatonin improved the development of 2-cell to 4-cell embryos in 24 hr. This was significantly better than in development of embryos in control group at the same time. The application of different doses of melatonin emphasis improved development of embryos at the 2-cell stage (4, 6). The concentration of melatonin positively affects the cleavage rate, but does not impact the number of blastocysts (11). As well as, blastocyst formation did not show any significant differences in the control and melatonin treated groups. Melatonin has been shown to increase the cleavage rate and developmental quality in mouse, buffalo and sheep embryos (3, 5, 6, 20). Melatonin supplementation to ewe embryo culture media have shown a dose dependent effects. Increasing of melatonin doses (10^-3^ M) causes high oxidative stress (21). However, low concenteration of melatonin is beneficial for embryo development (10^-9^M, 10^-6^M) (9, 11, 12, 21). As in this study the low dose of melatonin (10^-6^ nM) enhanced embryo development. There are controversial results on optimal using dose of melatonin for supplementation of culture media.

The number of dead cells was declined in blastocysts embryo after addition of melatonin to culture medium. This result has been confirmed in human embryos by inhibition of DNA transcription and decreases in lipid peroxidation (22). Actinomycin-D is known as a potent apoptotic inducer on different cell lines. It is used to inhibit DNA transcription (17). Although actinomycin-D and TNF-α increase apoptotic cells in the blastocyst, there are no effects on embryo development (14). Mura *et al* has shown that the addition of actinomycin-D to bovine embryo culture medium decreases the development and cleavage rates of embryos (23). This study demonstrated that actinomycin-D blocked embryo development at the 2-cell stage. If the embryos passed the developmental block, they would continue onto the blastocyst stage. Actinomycin-D increased the numbers of nonviable blastomere cells in the blastocysts, which confirms the results of other studies (23, 24). Melatonin supplementation has been shown to increase both the number and quality of bovine blastocysts by anti-apoptotic properties (9). In this study, melatonin improved both embryo development and decreased the number of nonviable blastomeres compared with embryos treated with actinomycin-D.

TNF-α, similar to actinomycin-D, retarded embryo development and increased the number of nonviable blastomere cells in blastocysts. Incubation of mouse and rat embryos with TNF-α has been shown to decrease embryo development (25-27). However, TNF-α has not increased the incidence of apoptosis in blastomeres compared to the control group. TNF-α might have an anti-proliferative effect on the embryo both in vitro and invivo (27). Our findings have supported the results of other studies (28). TNF-α may inhibit glucose consumption and cell proliferation in the rat blastocyst (29). TNF-α is naturally secreted as a cytokine during the early stages of pregnancy by the uterus (30). 

This cytokine assists with proliferation and continuation of pregnancy. However TNF-α has been shown to cause apoptosis of blastomers and a decline in embryo development. TNF-α (500 ng/ml) decreased protein synthesis in the morula and blastocyst stages (31). Developmental retardation of the embryo might be attributed to TNF-α. Supplementation of melatonin with TNF-α was not shown to inhibit its apoptotic properties. This might be due to the results of high doses of TNF-α or the delayed effects of melatonin in competitive with TNF-α. It seems the dose of melatonin which was used in this study was effective to inhibit the activity of actinomycine-D but it was not effective on TNF-α activity. It needs more study to clear that. 

## Conclusion

The present study is demonstrated that enriching the culture medium with melatonin enhanced the development rate and improved embryo quality. Melatonin could reduce and protect the embryo from the harmful effects of apoptotic inducers like actinomycin-D and TNF-α. Melatonin might be of clinical use in improving assisted reproductive technique efficiency.
